# The Association Between Respiratory Viruses and Asthma Exacerbation in Children Visiting Pediatric Emergency Department: A Retrospective Cohort Study

**DOI:** 10.3390/jcm14041311

**Published:** 2025-02-16

**Authors:** Won Seok Lee, Joo Young Song, Jeewon Shin, Sun Hee Choi, Man Yong Han, Kyung Suk Lee

**Affiliations:** 1 Department of Pediatrics, CHA Ilsan Medical Center, CHA University College of Medicine, Goyang 10414, Republic of Korea; leeerped@gmail.com (W.S.L.); joo08420@chamc.co.kr (J.Y.S.); a186030@chamc.co.kr (J.S.); 2 Department of Pediatrics, Kyung Hee University Hospital at Gangdong, Kyung Hee University College of Medicine, Seoul 05278, Republic of Korea; chsh0414@khnmc.or.kr; 3 Department of Pediatrics, CHA Bundang Medical Center, CHA University College of Medicine, Seongnam 13496, Republic of Korea; drmesh@gmail.com; 4 Department of Pediatrics, Hanyang University Guri Hospital, Hanyang University College of Medicine, Guri 11923, Republic of Korea

**Keywords:** asthma exacerbation, respiratory virus, rhinovirus, children, emergency department

## Abstract

**Background/Objectives:** Respiratory viral infections are a major cause of asthma exacerbations. However, studies examining the association between symptoms, signs, treatments, outcomes of asthma exacerbations, and various respiratory viruses in children are limited. This study aims to investigate the association between respiratory viral infections and clinical symptoms and signs, treatment, and hospital admission in children with asthma exacerbations visiting the pediatric emergency department. **Methods:** This study examined 395 children under 15 years of age who had a previous diagnosis of bronchial asthma, experienced asthma exacerbation, and visited an emergency center between 1 July 2015 and 30 June 2017. Among the 395 participants, respiratory virus polymerase chain reaction (PCR) was conducted in 96 patients (24.3%). The symptoms and signs of asthma exacerbation (dyspnea, tachypnea, chest retraction, wheezing, and gastrointestinal symptoms), treatment (oxygen supplementation and systemic steroid administration), symptom relief within 1 h, and hospital admission were analyzed. **Results:** Among the 96 patients who underwent respiratory virus PCR, at least one respiratory virus was detected in 72 (75.0%), and over two viruses were detected in 21 children (21.9%). Three common viruses were detected: rhinovirus in 59 (61.5%), adenovirus in 10 (10.4%), and respiratory syncytial virus (RSV) in nine children (9.4%). Rhinovirus infection was associated with tachypnea (adjusted odd ratio (aOR) 4.457, *p* = 0.007), chest retraction (aOR 3.142, *p* = 0.013), and systemic steroid administration (aOR 3.065, *p* = 0.034). Adenovirus infection was associated with oxygen supplementation via nasal cannula (aOR 5.297, *p* = 0.042). **Conclusions:** Rhinovirus was associated with tachypnea, chest retraction, and systemic steroid administration, while adenovirus was linked to oxygen supplementation in childhood asthma exacerbations. These findings will help clinicians to better observe asthma symptoms, select appropriate treatments, and improve outcomes for asthma exacerbations.

## 1. Introduction

Bronchial asthma is a common chronic inflammatory airway disease, and its prevalence and incidence are steadily increasing worldwide [1]. Asthma often begins in childhood, with a higher incidence in children than in adults [2]. A nationwide cross-sectional study in Korea, using data from 2016 to 2017, reported the following estimated asthma prevalence: 0.9% in infants, 2.3% in preschool children, 4.1% in school-aged children, 2.3% in adults, and 4.1% in older adults [3]. Asthma exacerbations are severe episodes in which symptoms, such as cough, shortness of breath, wheezing, and chest tightness, worsen significantly [4]. They occur across the spectrum of chronic severity, from intermittent asthma to severe persistent asthma, and they significantly contribute to the public health burden of the disease [4]. Asthma exacerbation is diagnosed based on a combination of symptoms, including episodes of progressive dyspnea, wheezing, chest tightness, and cough [4]. Although the exact pathophysiology of asthma associated with viral infections remains unclear, epidemiological findings suggest that respiratory infections may be involved in the onset and/or exacerbation of asthma [5,6]. Several respiratory viruses have been implicated, including human rhinovirus (hRV), respiratory syncytial virus (RSV), and human parainfluenza virus, all of which are associated with the onset and/or exacerbation of asthma [7]. Respiratory viral infections such as RSV and hRV can modify receptor expression and function, resulting in bronchoconstriction and persistent airway inflammation. Furthermore, viral infections in early childhood are associated with the onset of asthma, and genetic variations in ADRβ2 and TRPV1 may affect the disease severity and patient response to treatment [8]. hRV, the primary cause of the common cold, is clearly the most common single trigger for asthma exacerbations, accounting for up to 76% and 83% of exacerbations in children with wheezing and adults with asthma, respectively [9]. All studies to date suggest that the maintenance and homeostasis of the airway system are regulated by various immunological mediators, such as cytokines, which are produced by various cells in the airway [10]. Virus-induced asthma is thought to disrupt the maintenance/homeostasis of the airway system. However, the pleiotropic effects and redundancy of cytokines present major challenges in understanding the mechanisms underlying virus-induced asthma [11]. Although numerous studies have explored the pathogenesis and mechanism of virus-induced asthma, studies examining the association between the symptoms, signs, treatments, and outcomes of asthma exacerbations and various respiratory viral infections in children remain scarce. Therefore, this study aimed to evaluate the association between common respiratory viral infections, specifically hRV, adenovirus, and RSV, as detected by multiplex polymerase chain reaction (PCR) testing, and clinical symptoms and signs (e.g., dyspnea, tachypnea, chest retraction, wheezing, gastrointestinal symptoms), treatments (e.g., oxygen supplementation and systemic steroid administration), and outcomes (relief of symptoms within 1 h, hospitalization) in patients younger than 15 years with asthma exacerbations visiting a single pediatric ED over a 3-year period.

## 2. Materials and Methods

### 2.1. Participants and Definitions

We retrospectively analyzed the medical records of approximately 80,000 children and adolescents who visited a pediatric emergency center in South Korea between 1 July 2015 and 30 June 2017. This center specializes in providing urgent medical care to children and adolescents < 15 years of age. Upon review, 628 children were identified with asthma exacerbations based on International Classification of Disease (ICD) codes. Of these, 502 children were confirmed to have asthma, although our medical chart review met the clinical diagnostic criteria for childhood asthma. Childhood asthma was defined based on the following criteria: (1) age ≥ 2 and <15 years; (2) at least two claims under ICD-10 codes J45–J46; and (3) at least one claim during the baseline period for the prescription of asthma-related medications, including inhaled or systemic corticosteroids, bronchodilators, leukotriene receptor antagonists, and xanthine derivatives [1]. Furthermore, a detailed medical chart review confirmed that 395 children with a prior diagnosis of bronchial asthma met the clinical diagnostic criteria for asthma exacerbations. An asthma exacerbation is diagnosed by a combination of symptoms, such as an episode of progressive dyspnea, wheezing, chest tightness, and cough.

**Figure 1:**
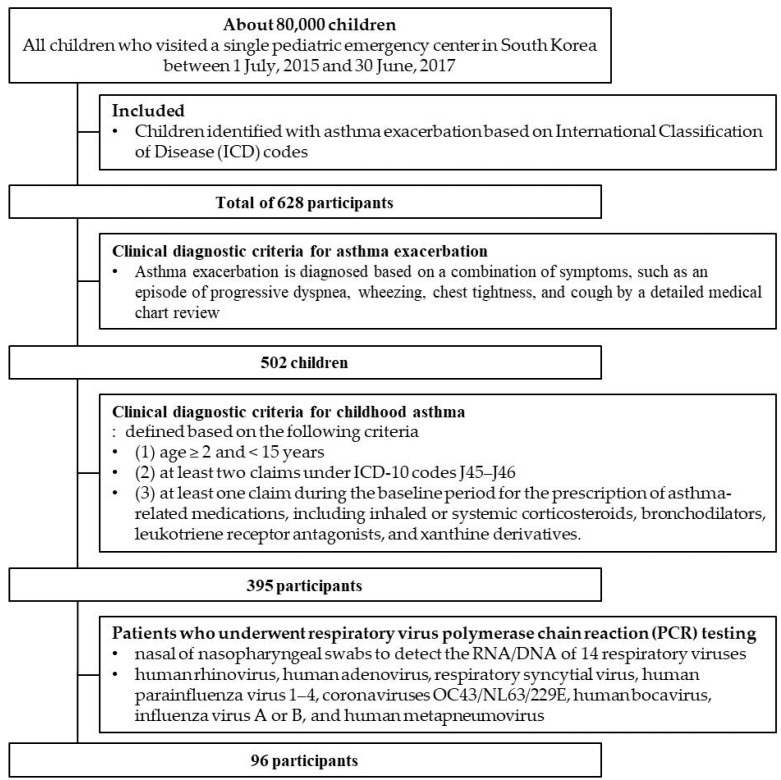
Study flow.

Among them, 96 patients underwent respiratory virus polymerase chain reaction (PCR) testing as part of the study (Figure 1). Multiplex PCR assays using nasal or nasopharyngeal (NP) swabs were performed on each respiratory sample to detect the RNA/DNA of 14 respiratory viruses, including hRV, human adenovirus, RSV, human parainfluenza virus 1–4, coronaviruses OC43/NL63/229E, human bocavirus, influenza virus A or B, and human metapneumovirus. The respiratory multiplex PCR consists of the following main steps that all occur in the same microwell [12]. Target viral cDNA is PCR amplified with a pair of virus-specific primers (1 h). One of the primers contains an iC at its 5′ terminus. The PCR product is labeled with a virus-specific EraCode tag (an eight-base oligonucleotide composed of both natural bases and iG) and an iG-biotin (biotinylated 2′-deoxy-iG triphosphate) with a target-specific extension (TSE) primer (30 min). The PCR step was carried out in an 8 μL reaction mixture containing 2 μL cDNA, 1 μL MC-PCR buffer, 0.16 μL of Taq polymerase, and 200 nM PCR forward and 5′-iC modified reverse primers under the following conditions: 5 min at 95 °C and 28 cycles of 5 s at 95 °C, 10 s at 55 °C, and 30 s at 72 °C. Both iG and iC are recognized by natural polymerase. iG and iC pair with each other but not with natural C and G. The tagged TSE product is captured by a color-addressed microsphere through the hybridization of its tag to its precise complementary oligonucleotide conjugated to the surface of the microsphere (10 min at room temperature). The captured product is labeled with fluorescent streptavidin–phycoerythrin (10 min). The fluorescent signal associated with each microsphere is measured in a Luminex LabMap 100 cytometer (1 h for a 96-well plate). A study reported that nasal-wash specimens that were positive for hRV, RSV, influenza virus, parainfluenza virus, or adenovirus by traditional techniques were reanalyzed using multiplex PCR, and all target viruses were detected with an overall sensitivity of 94% and specificity of 99% [12].

Children under 2 years of age were excluded owing to the frequent occurrence of other intrapulmonary airway disorders, such as bronchopulmonary dysplasia and acute viral bronchiolitis, which often complicate the definition and diagnosis in this age group [13]. Among childhood patients with asthma who met the diagnostic criteria outlined above, an asthma exacerbation was defined as a combination of symptoms—including progressive dyspnea, wheezing, chest tightness, and cough—observed in the ED by a pediatric emergency physician and confirmed through medical records.

This was a retrospective study, and informed consent was not required. The study was approved by the Institutional Review Board of CHA University Bundang CHA Hospital (IRB number 2018-09-031-003). All procedures were conducted in accordance with the relevant guidelines and regulations.

### 2.2. Characteristics, Allergen Sensitization, Treatments, and Outcomes

The clinical characteristics reviewed included sex, age, history of allergic disease, family history of allergic disease, laboratory test results (white blood cell (WBC) counts (per μL), eosinophil rates (%), C-reactive protein (mg/dL), and immunoglobulin E (IgE) levels), symptoms and signs, such as dyspnea, tachypnea [14], chest retraction, wheezing, and gastrointestinal symptoms (e.g., vomiting, abdominal pain, and diarrhea). The history of allergic diseases associated with anaphylaxis, such as bronchial asthma, allergic rhinitis, atopic dermatitis, food allergies, and anaphylaxis, was evaluated in the history and familial allergy history of each patient.

ImmunoCAP (Thermo Fisher, Uppsala, Sweden), MAST (AdvanSure AlloScreen, Seoul, Republic of Korea), and the skin prick test (SPT) were used to evaluate the allergen sensitization status of each patient. Most ImmunoCAP and MAST tests were conducted while patients were being treated for anaphylaxis in the ED. However, a few ImmunoCAP and MAST tests, alongside all SPTs, were conducted at the outpatient clinic following management in the ED. A positive specific allergen was defined according to criteria established in previous studies [15].

We analyzed treatments for asthma exacerbation, including oxygen supplementation (via nasal cannula, mask [simple, reservoir bag, or venture], high-flow nasal cannula), and systemic steroid administration in the ED. Additionally, we assessed treatment outcomes, such as symptom relief within 1 h and the need for hospital admission.

### 2.3. Primary and Secondary Outcomes

The primary outcomes were a comparison of clinical symptoms and signs (dyspnea, tachypnea, chest retraction, wheezing, and gastrointestinal symptoms), treatments (oxygen supplementation via nasal cannula, mask (simple, reservoir bag, or venturi), high-flow nasal cannula, systemic steroid administration), outcome (symptom relief within 1 h and hospital admission) in the ED for childhood asthma exacerbations. These comparisons were made between participants with no respiratory viruses detected and those with at least one virus detected, based on respiratory PCR results. We also analyzed the association between clinical symptoms and signs, treatments, and outcomes in participants with no respiratory virus detected versus those with more than two viruses detected.

The secondary outcomes focused on the association between various respiratory viral infections (hRV, adenovirus, and RSV) and clinical symptoms and signs, treatments, and outcomes in children with asthma exacerbation. We calculated crude odd ratios (cORs) and adjusted odd ratios (aORs) using logistic regression to analyze the secondary outcomes.

### 2.4. Statistical Analysis

Continuous variables, such as age, were expressed as interquartile ranges owing to their non-normal distribution. Results were statistically analyzed using the Mann–Whitney U-test and Fisher’s exact test. Logistic regression analysis was conducted to identify the risk factors for anaphylactic symptoms. *p*-values < 0.05 were considered statistically significant. All data were analyzed using SPSS version 27.0 software (IBM, Armonk, NY, USA).

## 3. Results

### 3.1. Seasonality and Clinical Characteristics

**Figure 2:**
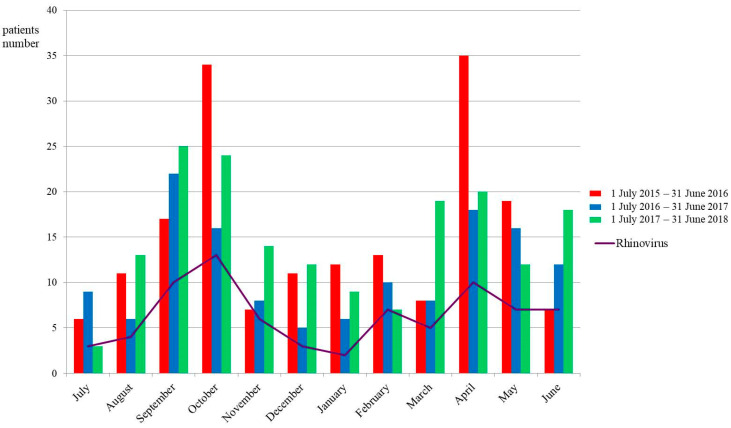
Monthly incidence of childhood asthma exacerbations and the number of cases in which human rhinovirus was detected. (N = 395). The bar chart displays the monthly number of acute asthma exacerbation patients visiting the pediatric emergency department, while the solid-line chart represents the number of patients with rhinovirus identified among acute asthma exacerbation cases. Notable seasonality was noted in the number of patients visiting the ED for childhood asthma exacerbation and in cases where human rhinovirus was detected. Peaks were observed in October and April, according to data from 1 July 2015 to 31 June 2018.

A notable seasonality was observed in the number of patients visiting the ED with childhood asthma exacerbation and the number of cases in which hRV was detected among them. Peaks occurred in October and April, based on data from 1 July 2015 to 31 June 2018. Figure 2 shows the monthly incidence of childhood asthma exacerbations and the number of cases with detected hRV. The clinical characteristics of all the patients are shown in Supplementary Table S1. Among the 395 patients with childhood asthma exacerbations, 258 (65.3%) were male, and the median age was 5.0 years (interquartile range: 3.0–8.0). Overall, 290 (73.4%) and 210 (53.2%) patients had a history of allergic diseases and a family history of allergic diseases, respectively. The symptoms and signs observed were dyspnea, tachypnea, chest retraction, and wheezing present in 343 (86.6%), 199 (50.4%), 154 (39.0%), and 367 (92.9%) patients, respectively. Oxygen and systemic steroids were administered to 93 (23.5%) and 181 (45.8%) patients, respectively. Symptom relief within 1 h in the ED was experienced by 265 (67.1%) patients. One hundred and twenty-five (31.6%) patients were admitted to the hospital for asthma exacerbations. Among the 395 participants, 96 (24.3%) patients with asthma exacerbation underwent respiratory PCR tests in the ED. No statistically significant differences were observed between participants with and without respiratory virus PCR tests regarding sex, age, allergic history, family history of allergies, laboratory test results, or allergen sensitization, except for food allergens. The symptoms and signs were dyspnea, tachypnea, and wheezing, present in 90 (93.8%), 199 (50.4%), and 367 (92.9%) patients, respectively. Significant differences in these symptoms and signs were observed between the participants who underwent respiratory virus PCR tests and those who did not. Additionally, oxygen and systemic steroids were administered to 93 (23.5%) and 181 (45.8%) patients, respectively, and 125 (31.6%) patients were admitted to the hospital for asthma exacerbations. Significant differences were observed in these treatments and outcomes in the ED between participants with and without respiratory virus PCR tests.

### 3.2. Primary Outcome

Among the 96 patients with asthma exacerbation who underwent respiratory PCR tests in the ED, no virus was detected in 24 (25.0%) patients, while at least one was detected in 72 (75.0%) patients, and more than two viruses were detected in 21 (21.9%). Wheezing was significantly associated with the detection of more than two viruses in childhood asthma exacerbation (*p* = 0.001). No significant associations were observed in symptoms and signs, excluding wheezing, oxygen supplementation, systemic steroids administration, symptom relief within 1 h, and hospital admission between patients with detected respiratory viruses and those without (Supplementary Table S2).

### 3.3. Secondary Outcome

The associations between common respiratory viral infections and clinical symptoms (dyspnea, tachypnea, chest retraction, wheezing, and gastrointestinal symptoms), treatments (oxygen supplementation via nasal cannula, mask, or high-flow nasal cannula, and systemic steroid administration), and outcomes (symptom relief within 1 h and hospital admission) were evaluated in children with asthma exacerbation who underwent respiratory virus PCR testing. (Supplementary Table S3) The three most common viruses detected in the respiratory virus PCR tests were hRV (59 children, 61.5%), adenovirus (10 children, 10.4%), and respiratory syncytial virus (9 children, 9.4%). hRV infection was associated with asthma exacerbation symptoms of tachypnea (50/59 children, 84.7%, *p* = 0.024), chest retraction (41/59 children, 69.5%, *p* = 0.022), and the need for systemic steroid administration (51/59 children, 86.4%, *p* = 0.027). Adenovirus infection was linked to the need for oxygen supplementation (*p* = 0.045) and oxygen treatment via nasal cannula (*p* = 0.027).

Other symptoms and signs, treatments, and outcomes of acute asthma exacerbation in children were not significantly associated with hRV, RSV, or adenovirus infections. Additionally, Supplementary Table S4 presents the association between symptoms, signs, treatment, and outcomes in childhood asthma exacerbation patients with less commonly detected respiratory viruses (parainfluenza virus, coronavirus OC43/NL63/229E, human bocavirus, influenza virus, and human metapneumovirus).

Logistic regression analysis revealed consistent results. hRV infection was significantly associated with the symptom of tachypnea (adjusted odd ratio (aOR) 4.457, *p* = 0.007) and chest retraction (aOR 3.142, *p* = 0.013), as well as with systemic steroid administration (aOR 3.065, *p* = 0.034) in children with asthma exacerbation. In addition, adenovirus infection was associated with the need for oxygen supplementation (aOR 5.574, *p* = 0.041) and oxygen treatment via nasal cannula (aOR 5.297, *p* = 0.042) in children with asthma exacerbation (Supplementary Table S5).

## 4. Discussion

This study evaluated the relationship between common respiratory viral infections—especially hRV, adenovirus, and RSV—and clinical symptoms, signs, treatments, and outcomes in children with asthma exacerbation who visited the ED. We found that hRV infection was associated with symptoms of tachypnea and chest retraction, as well as the use of systemic steroid administration, while adenovirus infection was associated with the need for oxygen supplementation via nasal cannula in children experiencing asthma exacerbation. To our knowledge, this study is one of the few to investigate the association between the symptoms and signs, treatments, and outcomes of asthma exacerbations, and various respiratory viral infections in children. These findings highlight the significant influence of respiratory viral infections on asthma exacerbations in children. Therefore, clinicians should closely monitor respiratory rates and the degree of chest retraction in children with asthma exacerbations identified as having an hRV infection in the ED. They could also anticipate the need for systemic steroid administration and provide it promptly, which may improve the treatment outcomes for these patients. Similarly, clinicians managing children with asthma exacerbation identified as having an adenovirus infection could carefully monitor oxygen saturation levels and promptly administer oxygen before the onset of significant hypoxia. This proactive approach can significantly contribute to symptom relief and improve clinical outcomes in ED.

Asthma can be triggered by diverse factors, including allergen exposure, exercise, irritants, weather changes, and viral infections. Acute worsening of symptoms, termed exacerbation, often requires additional respiratory medications or hospitalization and can be potentially life-threatening [16]. Respiratory tract infections are a major trigger for asthma exacerbations, accounting for up to 90% of these acute episodes [17]. The pathophysiology of asthma is closely linked to viral exposure and immune responses [16]. Although the epidemiology and immunopathology of respiratory viral infections in asthma are extensively studied, the extent to which asthma predisposes individuals to viral infections remains unclear.

Epidemiological studies with sensitive detection methods, such as PCR, show respiratory viral infection as a significant trigger of asthma exacerbations, especially in spring and fall. Viral infections account for 85–95% of asthma exacerbations in children and up to 80% in adults [18]. hRV is frequently detected in these acute conditions, but other respiratory viruses identified during exacerbations include adenovirus, bocavirus, coronavirus, cytomegalovirus, enterovirus, herpes simplex virus, influenza virus, metapneumovirus, parainfluenza virus, and RSV [16,19]. hRV was the most frequently identified virus across all ages, with a prevalence of 45.7% in children and 31.1% in adults, while other respiratory viruses showed age-dependent distribution patterns. In children, RSV has a prevalence of 17.7%, enterovirus 11.8%, coronavirus 8.4%, and influenza virus 7.4% [20]. Viral coinfection occurs frequently in 10–40% of cases and is more common in young children [20]. In our study, at least one respiratory virus was detected in 75% of the patients, and 61.5% were found to be infected with rhinovirus. Although this result appears to show a slightly lower viral detection rate compared to the studies mentioned above, it is consistent with the context that viral infections contribute the most to asthma exacerbations, with rhinovirus accounting for the highest proportion.

Viral clearance and infection resolution require a multifaceted response, starting with the innate immune system and progressing to adaptive immune responses. The innate immune system serves as the first line of defense and also modulates immune-inflammatory responses [21]. Disruptions in this balance impair the innate immune response and trigger abnormal inflammatory reactions, creating a vicious cycle [21]. Studies show that a disrupted innate immune response in asthma may increase the severity of respiratory infections. However, whether this abnormal response is an inherent characteristic of asthma or a consequence of its interaction with the inflammatory environment remains unclear.

hRVs are the most common respiratory viruses linked to asthma exacerbations, making respiratory viruses a key focus of research on acute events. hRVs are non-enveloped, positive-strand RNA viruses belonging to the *Picornaviridae* family and genus *Enterovirus*, classified into three species (hRV-A, hRV-B, and hRV-C) [22]. With over 160 distinct hRV genotypes, the structural and genetic variability of hRVs has hindered efforts to develop effective antivirals. PCR is the preferred method for identifying hRVs in nasal mucus samples. Up to 35% of asymptomatic individuals test positive; however, the virus does not cause chronic infection or ‘‘colonization’’ in healthy individuals [23]. hRV circulates year-round with multiple coexisting genotypes, peaking in early autumn and late spring in temperate climates [24]. In our study, a seasonal pattern was observed in asthma exacerbations among children and in the detection of hRV cases within this group, with the peaks observed in October and April.

hRV infection ranges from mild upper respiratory tract illnesses to severe lower respiratory tract illnesses and is linked to bronchiolitis, asthma, and chronic obstructive pulmonary exacerbations [25]. The Childhood Origins of Asthma (COAST) study shows that children who experienced wheezing due to hRV within their first 3 years face a significantly higher risk of developing asthma by age 6 (OR 9.8) compared to those with RSV-induced wheezing (OR 2.6) [26]. Additionally, 90% of children with hRV-induced wheezing in their third year develop asthma at age 6 (OR 26) [27].

This study showed that hRV infection was significantly associated with acute symptoms such as tachypnea and chest retractions, along with a greater need for systemic steroid treatment in the ED. Several experimental models have been developed to investigate the relationship between hRV infections and asthma exacerbations. In vitro models demonstrate that hRV infection in the lower airway epithelium induces Intercellular Adhesion Molecule-1 (ICAM-1) expression. ICAM-1, the cellular receptor of the major hRV group, promotes both inflammatory cell infiltration and hRV infection [28]. A human model of virus-induced asthma exacerbations uses hRV type 16 inoculation in the nostrils of patients with asthma and healthy volunteers. Their study also shows a human model of hRV infection in patients with mild asthma, demonstrating that hRV infection worsens respiratory symptoms, induces bronchial hyper-responsiveness, and impairs lung function [29]. Immunological changes include an increased Th2 response, along with deficient IL-10 and an atypical Th1 response [29]. The epithelium of patients with asthma is more fragile than that of healthy individuals with compromised barrier function that allows the leakage of harmful agents, including allergens, and pathogens, into the airway mucosa [30,31]. hRV binds to various cell types, primarily targeting bronchial epithelial cells, which serve as the main hosts for viral replication [30,31]. This shows that an impaired innate immune response is linked to increased viral replication, suggesting it may underlie the heightened susceptibility of patients with asthma to viral infections.

Furthermore, upregulated T2 immune pathways characterize the main inflammatory phenotype observed in asthma, with levels closely linked to disease severity and management [32]. RV has been demonstrated to trigger various factors linked to this pathway, including the activation of the pivotal transcription factor STAT6, stimulation of T cell and eosinophil chemokines CCL11, CCL17, CCL22, CCL26, epithelial-derived IL-25 and IL-33, and cytokines IL-4, IL-5, IL-13, and prostaglandin D2 [33]. Early in the T2 cascade, IL-33 (produced in reaction to bronchial epithelial infection) seems to be a key mediator in both TH2 cells and T2 innate lymphoid cell (ILC2)-driven responses to RV [34]. ILC2 serves as a significant source of T2 cytokines such as IL-5, IL-9, and IL-13, promoting the recruitment and activation of eosinophils and mast cells, as well as goblet cell-mediated mucus production [33]. Airway neutrophilia also increases during virus-induced exacerbations and may play a crucial role in the pathogenesis of exacerbation by activating neutrophil extracellular traps [33]. RSV infections are frequently linked to asthma exacerbations in children, young adults, and the elderly [16]. hRV and RSV primarily spread through direct contact and aerosol particles. They replicate in the ciliated epithelial cells of the upper respiratory tract and the medium-sized to large-sized airways of the lower respiratory tract [33]. Epidemics typically occur in midwinter, and nearly all children have had an RSV infection at 2 years [34]. RSV is a *pneumovirus* from the *Paramyxoviridae* family, consisting of a single-stranded, enveloped RNA virus with two main antigenic groups, A and B [34]. The genetic diversity of proteins within the A and B RSV groups has led to the formation of several subgroups, with 10 and 13 genotypes in groups A and B, respectively [34]. The RSV A subtype causes the most severe manifestations of RSV infection, such as fever, severe cough, wheezing breath, dyspnea, and cyanosis [16]. Several prospective long-term follow-up studies show that RSV-induced bronchiolitis is associated with the later development of asthma [35,36]. RSV induces apoptosis and necrosis in epithelial cells, causing greater damage to the airway epithelium than hRV [34]. Upon contact with immune cells, RSV does not trigger Th1 signaling but instead induces the production of cytokines and chemokines, such as IL-4 and IL-17, leading to a Th2 response [37]. The role of RSV infection in asthma extends beyond asthma exacerbation; its contribution to the onset of the disease has been proposed and disputed [38]. Otherwise, adenovirus is implicated in some cases of near-fatal events of asthma exacerbations [39].

However, this study has some limitations. First, the retrospective study design makes it difficult to establish a clear causal relationship. In addition, the sample size was small. The pediatric emergency center where our study was conducted is a medical institution that treated approximately 80,000 children over the three-year study period. However, the sample size inevitably became smaller as we filtered out patients who met the precise diagnostic criteria for acute asthma exacerbation and further selected only those who underwent respiratory virus PCR testing. To address the limitations of our retrospective study and the small sample size, we are considering analyzing a larger sample over an extended study period in future research. Second, hRV can be detected in the respiratory epithelial cells or secretions of asymptomatic healthy individuals. Since children spend more time in group settings, the likelihood of false positives may be higher compared to that of adults. This potential source of error was not addressed in this study. Finally, among the participants included in our study, only 96 (24.3%) children underwent PCR testing, while the remaining patients did not undergo PCR tests performed in the ED. The ED is a clinical setting in which diagnosis and treatment must be delivered urgently. Moreover, performing nasal/nasopharyngeal swabs is particularly challenging because the procedure can cause discomfort or pain, especially in young children. The high cost of PCR testing is another factor that must be considered by physicians and patients. As a result, PCR tests were more likely to be performed in children with relatively severe symptoms or those requiring hospitalization.

However, the association between symptoms, signs, treatment, and outcomes of asthma exacerbations in children and various respiratory viral infections was investigated in this study, an area with limited existing research. Furthermore, viral infections play a more significant role in asthma exacerbations in children compared to those in adults. Therefore, the findings on the role of viruses in pediatric asthma exacerbations are valuable for clinicians in predicting symptoms and determining treatment for patients with asthma.

Human metapneumovirus (hMPV) is a single-stranded RNA virus belonging to the Pneumoviridae subfamily that causes wheezing and lower respiratory tract symptoms, such as RSV [40]. Approximately 5% of patients experiencing asthma exacerbation have hMPV detected globally, compared to a significantly higher percentage of individuals with hMPV-related lower respiratory tract infections who have been diagnosed with asthma [41]. Children with hMPV-related lower respiratory tract infections were included in a study in the United States, in which 14–33% had an asthma diagnosis or a history of wheezing [41]. Animal models have shown that airway hyper-responsiveness and inflammation are induced following hMPV infection and that hMPV can persist in vivo by suppressing innate immune responses and inducing abnormal adaptive responses.

Additionally, the SARS-CoV-2 pandemic and subsequent implementation of non-pharmaceutical interventions (e.g., mask-wearing, social distancing, and cessation of international travel) reduced the transmission of some viral respiratory pathogens [42]. In the United States, rhinovirus and enterovirus circulation decreased in March 2020, remained low until May 2020, and then rose to near-pre-pandemic seasonal levels [43]. The circulation of RSV also decreased in early 2020 and did not increase until spring 2021, while adenovirus circulated at lower levels throughout 2020 and as of early May 2021 in the United States [44].

## 5. Conclusions

In conclusion, the association between common respiratory viral infections, such as hRV, adenovirus, and RSV, with clinical symptoms and signs, treatment, and treatment outcomes in children with asthma exacerbations was assessed. We found that hRV infection was associated with symptoms of tachypnea, chest retraction, and systemic steroid administration, while adenovirus infection was linked to oxygen supplementation via nasal cannula in children with asthma exacerbations. Since the SARS-CoV-2 pandemic, the global prevalence of respiratory viruses has shifted. Given these changes in viral epidemiology, further research is needed to understand the effect of viruses on asthma exacerbations in children. Such efforts will help clinicians in emergency settings better observe asthma symptoms and signs, select appropriate treatments, and administer them promptly, ultimately improving outcomes for children with asthma exacerbations.

## Data Availability

The datasets used during the current study are available from the corresponding author on reasonable request.

## Supplementary Materials

The following supporting information can be downloaded at: https://www.mdpi.com/article/10.3390/jcm14041311/s1, Table S1: Clinical characteristics of children with asthma exacerbation visiting the pediatric emergency department (N = 395); Table S2: Association between respiratory virus detection and clinical symptoms, signs, treatments, and outcomes in children with asthma exacerbations visiting the pediatric emergency department (N = 96); Table S3: Association of the various respiratory viral infections with clinical symptoms, signs, treatments, and outcomes in children with asthma exacerbation (N = 96); Table S4: Association of other respiratory virus infections with clinical symptoms, signs, treatments, and outcomes in children experiencing asthma exacerbation; Table S5: Logistic regression analysis of the association between respiratory virus and clinical symptoms, signs, treatments, and outcomes in children with asthma exacerbations (N = 96).

## Abbreviation

ED	emergency department

## References

[1] Lee W.S., Hwang J.K., Ryu J., Choi Y.J., Oh J.W., Kim C.R., Han M.Y., Oh I.H., Lee K.S. (2023). The relationship between childhood asthma and socioeconomic status: A Korean nationwide population-based study. Front. Public Health.

[2] Dharmage S.C., Perret J.L., Custovic A. (2019). Epidemiology of Asthma in Children and Adults. Front. Pediatr..

[3] Ha J., Lee S.W., Yon D.K. (2020). Ten-year trends and prevalence of asthma, allergic rhinitis, and atopic dermatitis among the Korean population, 2008–2017. Clin. Exp. Pediatr..

[4] Hasegawa K., Craig S.S., Teach S.J., Camargo C.A. (2021). Management of Asthma Exacerbations in the Emergency Department. J. Allergy Clin. Immunol. Pract..

[5] Kusel M.M., Klerk N.H.d., Kebadze T., Vohma V., Holt P.G., Johnston S.L., Sly P.D. (2007). Early-life respiratory viral infections, atopic sensitization, and risk of subsequent development of persistent asthma. J. Allergy Clin. Immunol..

[6] Johnston N.W., Sears M.R. (2006). Asthma exacerbations. 1: Epidemiology. Thorax.

[7] Hayashi Y., Sada M., Shirai T., Okayama K., Kimura R., Kondo M., Okodo M., Tsugawa T., Ryo A., Kimura H. (2022). Rhinovirus Infection and Virus-Induced Asthma. Viruses.

[8] Manti S., Gambadauro A., Galletta F., Ruggeri P., Piedimonte G. (2024). Update on the Role of β2AR and TRPV1 in Respiratory Diseases. Int. J. Mol. Sci..

[9] Turunen R., Koistinen A., Vuorinen T., Arku B., Söderlund-Venermo M., Ruuskanen O., Jartti T. (2014). The first wheezing episode: Respiratory virus etiology, atopic characteristics, and illness severity. Pediatr. Allergy Immunol..

[10] Toews G.B. (2001). Cytokines and the lung. Eur. Respir. J. Suppl..

[11] Gern J.E. (2003). Mechanisms of virus-induced asthma. J. Pediatr..

[12] Lee W.M., Grindle K., Pappas T., Marshall D.J., Moser M.J., Beaty E.L., Shult P.A., Prudent J.R., Gern J.E. (2007). High-Throughput, Sensitive, and Accurate Multiplex PCR-Microsphere Flow Cytometry System for Large-Scale Comprehensive Detection of Respiratory Viruses. J. Clin. Microbiol..

[13] Moral L., Vizmanos G., Torres-Borrego J., Praena-Crespo M., Tortajada-Girbés M., Pellegrini F.J., Assensio Ó. (2019). Asthma diagnosis in infants and preschool children: A systematic review of clinical guidelines. Allergol. Immunopathol..

[14] Kleinman M.E., Chameides L., Schexnayder S.M., Samson R.A., Hazinski M.F., Atkins D.L., Berg M.D., Caen A.R.d., Fink E.L., Freid E.B. (2010). Part 14: Pediatric advanced life support: 2010 American Heart Association Guidelines for Cardiopulmonary Resuscitation and Emergency Cardiovascular Care. Circulation.

[15] Bernstein I.L., Li J.T., Bernstein D.I., Hamilton R., Spector S.L., Tan R., Sicherer S., Golden D.B.K., Khan D.A., Nicklas R.A. (2008). Allergy diagnostic testing: An updated practice parameter. Ann. Allergy Asthma Immunol..

[16] Urbani F., Cometa M., Martelli C., Santoli F., Rana R., Ursitti A., Bonato M., Baraldo S., Contoli M., Alberto P. (2023). Update on virus-induced asthma exacerbations. Expert. Rev. Clin. Immunol..

[17] Corne J.M., Marchal C., Smith S., Schreiber J., Sanderson G., Holgate S.T., Johnston S.L. (2002). Frequency, severity, and duration of rhinovirus infections in asthmatic and non-asthmatic individuals: A longitudinal cohort study. Lancet.

[18] Jartti T., Bønnelykke K., Elenius V., Feleszko W. (2020). Role of viruses in asthma. Semin. Immunopathol..

[19] Johnston S.L., Pattemore P.K., Sanderson G., Smith S., Lampe F., Josephs L., Symington P., O’Toole S., Myint S.H., Tyrrell D.A. (1995). Community study of role of viral infections in exacerbations of asthma in 9–11 year old children. BMJ.

[20] Zheng X.Y., Xu Y.J., Gaun W.J., Lin L.F. (2018). Regional, age and respiratory-secretion-specific prevalence of respiratory viruses associated with asthma exacerbation: A literature review. Arch. Virol..

[21] Kabata H., Moro K., Koyasu S. (2018). The group 2 innate lymphoid cell (ILC2) regulatory network and its underlying mechanisms. Immunol. Rev..

[22] Turner R.B., Lee W.M., Richman D.D., Whitley R.J., Hayden F.G. (2017). Rhinovirus. Clinical Virology.

[23] Jartti T., Gern J.E. (2011). Rhinovirus-associated wheeze during infancy and asthma development. Curr. Respir. Med. Rev..

[24] Jartti T., Lee W.M., Papas T., Evans M., Lemanske R.F., Gern J.E. (2008). Serial viral infections in infants with recurrent respiratory illnesses. Eur. Respir. J..

[25] Greenberg S.B. (2003). Respiratory consequences of rhinovirus infection. Arch. Intern. Med..

[26] Jackson D.J., Gangnon E.R., Evans M.D., Roberg K.A., Anderson E.L., Papas T.E., Printz M.C., Lee W.M., Shult P.A., Reisdorf E. (2008). Wheezing rhinovirus illnesses in early life predict asthma development in high-risk children. Am. J. Respir. Crit. Care Med..

[27] Lemanske R.F., Jackson D.J., Gangnon R.E., Evans M.D., Li Z., Shult P.A., Kirk C.J., Reisdorf E., Roberg K.A., Anderson E.L. (2005). Rhinovirus illnesses during infancy predict subsequent childhood wheezing. J. Allergy Clin. Immunol..

[28] Saturni S., Contoli M., Spanevello A., Papi A. (2015). Models of Respiratory Infections: Virus-Induced Asthma Exacerbations and Beyond. Allergy Asthma Immunol. Res..

[29] Message S.D., Laza-Stanca V., Mallia P., Parker H.L., Zhu J., Kebadze T., Contoli M., Sanderson G., Kon O.M., Papi A. (2008). Rhinovirus-induced lower respiratory illness is increased in asthma and related to virus load and Th1/2 cytokine and IL-10 production. Proc. Natl. Acad. Sci. USA.

[30] Barbato A., Turato G., Baraldo S., Bazzan E., Calabrese F., Panizzolo C., Zanin M.E., Zuin R., Maestrelli P., Fabbri L.M. (2006). Epithelial damage and angiogenesis in the airways of children with asthma. Am. J. Respir. Crit. Care Med..

[31] Hammad H., Lambrecht B.N. (2015). Barrier Epithelial Cells and the Control of Type 2 Immunity. Immunity.

[32] Jackson D.J., Makrinioti H.M., Rana B.M., Shamji B.W., Trujillo-Torralbo M.B., Footitt J., Del-Rosario J., Telcian A.G., Nikonova A., Zhu J. (2014). IL-33- dependent type 2 inflammation during rhinovirus-induced asthma exacerbations in vivo. Am. J. Respir. Crit. Care Med..

[33] Mosser A.G., Vrtis R., Burchell L., Lee W.M., Dick C.R., Weisshaar E., Bock D., Swenson C.A., Cornwell R.D., Meyer K.C. (2005). Quantitative and qualitative analysis of rhinovirus infection in bronchial tissues. Am. J. Respir. Crit. Care Med..

[34] Jartti T., Gern J.E. (2017). Role of viral infections in the development and exacerbation of asthma in children. J. Allergy Clin. Immunol..

[35] Ruotsalainen M., Hyvärinen M.K., Piippo-Savolainen E., Korppi M. (2013). Adolescent asthma after rhinovirus and respiratory syncytial virus bronchiolitis. Pediatr. Pulmonol..

[36] Henderson J., Hilliard T.N., Sherriff A., Stalker D., Shammari N.A., Thomas H.M. (2005). Hospitalization for RSV bronchiolitis before 12 months of age and subsequent asthma, atopy and wheeze: A longitudinal birth cohort study. Pediatr. Allergy Immunol..

[37] Mukherjee S., Lindell D.M., Berlin A.A., Morris S.B., Shanley T.P., Hershenson M.B., Lukacs N.W. (2011). IL-17-induced pulmonary pathogenesis during respiratory viral infection and exacerbation of allergic disease. Am. J. Pathol..

[38] Sigurs N., Bjarnason R., Sigurbergsson F., Kjellman B. (2000). Respiratory syncytial virus bronchiolitis in infancy is an important risk factor for asthma and allergy at age 7. Am. J. Respir. Crit. Care Med..

[39] Papadopoulos N.G., Johnston S.L. (1998). Viruses and asthma exacerbations. Thorax.

[40] Coverstone A.M., Want L., Sumino K. (2019). Beyond Respiratory Syncytial Virus and Rhinovirus in the Pathogenesis and Exacerbation of Asthma. Immunol. Allergy Clin. N. Am..

[41] Bakakos A., Sotiropoulou Z., Vontetsianos A., Zaneli S., Papaioannou A.I., Bakakos P. (2023). Epidemiology and Immunopathogenesis of Virus Associated Asthma Exacerbations. J. Asthma Allergy.

[42] Rudd P.A., Thomas B.J., Zaid A., MacDonald M., Kan-O K., Rolph M.S., Soorneedi A.R., Bardin P.G., Mahalingam S. (2017). Role of human metapneumovirus and respiratory syncytial virus in asthma exacerbations: Where are we now?. Clin. Sci..

[43] Haddadin Z., Schuster J.E., Spieker A.J., Rahman H., Blozinski A., Stewart L., Campbell A.P., Lively J.Y., Michaels M.G., Williams J.V. (2021). Acute respiratory illnesses in children in the SARS-CoV-2 pandemic: Prospective multicenter study. Pediatrics.

[44] Olsen S.J., Winn A.K., Budd A.P., Prill M.M., Stell J., Midgley C.M., Kniss K., Burns E., Rowe T., Foust A. (2021). Changes in Influenza and Other Respiratory Virus Activity During the COVID-19 Pandemic—United States, 2020–2021. MMWR Morb. Mortal. Wkly. Rep..

