# Supplementary material for: The Association Between Respiratory Viruses and Asthma Exacerbation in Children Visiting Pediatric Emergency Department: A Retrospective Cohort Study
Source: J Clin Med. 2025 Feb 16;14(4):1311. doi: 10.3390/jcm14041311 (PMC11856561; doi:10.3390/jcm14041311)
Supplement: Supplementary file 1 [file jcm-14-01311-s001.zip › jcm-3382647-supplementary.pdf]

Journal of
Clinical Medicine

MDPI

Supplementary Table S1. Clinical characteristics of children with asthma exacerbation visiting the pediatric emergency department (N = 395)

Copyright: © 2025 by the authors. Licensee MDPI, Basel, Switzerland. This article is an open access article distributed under the terms and conditions of the Creative Commons Attribution (CC BY) license (https://creativecommons.org/licenses/by/4.0/).

	Total participants (N=395)	Participants with respiratory virus PCR results (N=96)	Participants without respiratory virus PCR results (N=299)	P value
Sex (boy, n, %)	258 (65.3)	57 (59.4)	201 (67.2)	0.160
Age (median, yr)	5.0 (3.0-8.0)	4.0 (3.0-7.0)	5.0 (3.0-8.0)	0.250
All allergic history (n, %)	290 (73.4)	65 (67.7)	225 (75.3)	0.146
Bronchial asthma (n, %)	210 (53.2)	53 (55.2)	157 (52.5)	0.645
Allergic rhinitis (n, %)	154 (39.0)	29 (30.2)	125 (41.8)	0.054
Atopic dermatitis (n, %)	81 (21.0)	16 (16.7)	67 (22.4)	0.230
Food allergy (n, %)	40 (10.1)	9 (9.4)	31 (10.4)	0.779
Anaphylaxis (n, %)	1 (0.3)	0 (0.0)	1 (0.3)	0.570
Familial allergy histories [1)]	210 (53.2)	55 (57.3)	155 (51.8)	0.352
WBC (median, /uL) †	12,245 (9,128–15,658)	13,050 (9760–16,110)	11,110 (8,340–14,830)	0.550
Eonisnophil (median, %) †	2.75 (1.00–5.30)	2.30 (0.90–4.90)	3.10 (1.00–5.80)	0.178
C-reactive protein (median, mg/dL) †	0.62 (0.16–1.30)	0.76 (0.19–1.32)	0.50 (0.13–1.28)	0.623
Total IgE (median, U/mL) †	206.4 (60.9–535.3)	206.4 (87.4–476.7)	224.4 (54.3–558.0)	0.500
Allergen sensitization [2)]				
All allergens (n, %) (N=262)	213 (81.3)	69 (83.1)	144 (80.4)	0.604
DP or DF (n, %) (N = 256)	160 (62.5)	49 (60.5)	111 (63.4)	0.652
Tree, grass allergen (n, %) (N = 256)	76 (29.7)	22 (27.2)	54 (30.9)	0.547
Animal allergen (n, %) (N = 256)	89 (34.8%)	25 (30.9)	64 (36.6)	0.373
Fungus allergen (n, %) (N = 256)	22 (8.6%)	7 (8.6)	15 (8.6)	0.985
Food allergen (n, %) (N = 256)	89 (34.8%)	39 (48.1)	50 (28.6)	0.002*

 https://doi.org/10.3390/jcm14041311

Symptoms and sign				
Dyspnea (n, %)	343 (86.6)	90 (93.8)	253 (84.6)	0.021*
Tachypnea (n, %)	199 (50.4)	74 (77.1)	125 (41.8)	< 0.001*
Chest retraction (n, %)	154 (39.0)	58 (60.4)	96 (32.1)	< 0.001*
Wheezing (n, %)	367 (92.9)	93 (96.9)	274 (91.6)	0.082
Gastrointestinal symptoms 3) (n, %)	22 (5.6)	5 (5.2)	17 (5.7)	0.859
Oxygen supplementation (n, %)	93 (23.5)	48 (50.0)	45 (15.1)	< 0.001*
Nasal cannula (n, %)	86 (21.8)	45 (46.9)	41 (13.7)	< 0.001*
Mask (simple, reservoir bag, or venturi) (n, %)	5 (1.3)	4 (4.2)	1 (0.3)	0.003*
High-flow nasal cannula (n, %)	4 (1.0	2 (2.1)	2 (0.7)	0.228
Systemic steroid administration (n, %)	181 (45.8)	76 (79.2)	105 (35.1)	< 0.001*
Symptom relieved within 1 h (n, %)	265 (67.1)	4 (4.2)	261 (87.3)	< 0.001*
Admission to hospital (n, %)	125 (31.6)	77 (80.2)	49 (16.4)	< 0.001*

Abbreviations: PCR, polymerase chain reaction; WBC, white blood cell; DP, D. ; DF, D.

1) Familial allergy histories include bronchial asthma, allergic rhinitis, atopic dermatitis, food allergy, anaphylaxis, and other allergic disease such as allergy to drugs, sunlight, etc.

2) Allergen sensitization was defined as at least one positive result from the immune CAP, MAST, and skin prick test.

3) Gastrointestinal symptoms include vomiting, abdominal pain, and diarrhea.

* indicates a P value of < 0.05, and † indicates interquartile range

Supplementary Table S2. Association between respiratory virus detection and clinical symptoms, signs, treatments, and outcomes in children with asthma exacerbations visiting the pediatric emergency department (N = 96)

Clinical symptoms, signs, treatments, and outcomes	Participants with respiratory virus PCR results (N = 96)	Virus not detected (n, %), (n = 24)	At least one virus detected (n = 72)		More than two viruses detected (n = 21)	
			n (%)	P value	n (%)	P value
Symptoms and signs						
Dyspnea (n, %)	90 (93.8)	23 (95.8)	67 (93.1)	0.626	19 (90.5)	0.483
Tachypnea (n, %)	74 (77.1)	15 (62.5)	59 (81.9)	0.050	16 (76.2)	0.912
Chest retraction (n, %)	58 (60.4)	11 (45.8)	47 (65.3)	0.092	15 (71.4)	0.243
Wheezing (n, %)	93 (96.9)	24 (100.0)	69 (95.8)	0.310	18 (85.7)	0.001*
Gastrointestinal symptoms [1] (n, %)	5 (5.2)	2 (8.3)	3 (4.2)	0.426	1 (4.8)	0.917
Oxygen supplementation (n, %)	48 (50.0)	10 (41.7)	38 (52.8)	0.346	9 (42.9)	0.457
Nasal cannula (n, %)	45 (46.9)	9 (37.5)	36 (50.0)	0.288	9 (42.9)	0.676
Mask (simple, reservoir bag, or venturi) (n, %)	4 (4.2)	2 (8.3)	2 (2.8)	0.238	1 (4.8)	0.877
High-flow nasal cannula (n, %)	2 (2.1)	0 (0.0)	2 (2.8)	0.409	0 (0.0)	-
Systemic steroid administration (n, %)	76 (79.2)	18 (75.0)	58 (80.6)	0.562	16 (76.2)	0.704
Symptom relief within 1 h (n, %)	4 (4.2)	2 (8.3)	2 (2.8)	0.238	0 (0.0)	-
Admission to hospital (n, %)	77 (80.2)	20 (83.3)	57 (79.1)	0.657	14 (66.7)	0.078

Abbreviations: PCR, polymerase chain reaction.

[1] Gastrointestinal symptoms include vomiting, abdominal pain, and diarrhea.

* indicates a P value of < 0.05, and † indicates interquartile range

Supplementary Table S3. Association of the various respiratory viral infections with clinical symptoms, signs, treatments, and outcomes in children with asthma exacerbation (N = 96)

Clinical symptoms and signs, treatments, outcomes	Virus not detected (n = 24)	Rhinovirus		Adenovirus		RSV	
		(n, %)	P value	(n, %)	P value	(n, %)	P value
Positive in PCR test (n/N, %)		59/96 (61.5)		10/96 (10.4)		9/96 (9.4)	
Symptoms and signs							
Dyspnea (n, %)	23 (95.8)	57 (96.6)	0.144	9 (90.0)	0.605	8 (88.9)	0.527
Tachypnea (n, %)	15 (62.5)	50 (84.7)	0.024*	8 (80.0)	0.817	6 (66.7)	0.435
Chest retraction (n, %)	11 (45.8)	41 (69.5)	0.022*	8 (80.0)	0.181	3 (33.3)	0.081
Wheezing (n, %)	24 (100.0)	57 (96.6)	0.851	9 (90.0)	0.187	9 (100.0)	0.571
Gastrointestinal symptoms [1)] (n, %)	2 (8.3)	3 (5.1)	0.945	0 (0.0)	-	0 (0.0)	-
Oxygen supplementation (n, %)	10 (41.7)	32 (54.2)	0.294	8 (80.0)	0.045*	3 (33.3)	0.294
Nasal cannula (n, %)	9 (37.5)	30 (50.8)	0.325	8 (80.0)	0.027*	3 (33.3)	0.392
Mask (simple, reservoir bag, venturi) (n, %)	2 (8.3)	2 (3.4)	0.631	1 (10.0)	0.329	0 (0.0)	-
High-flow nasal cannula (n, %)	0 (0.0)	2 (3.4)	0.258	0 (0.0)	-	0 (0.0)	-
Systemic steroid administration (n, %)	18 (75.0)	51 (86.4)	0.027*	8 (80.0)	0.945	5 (55.5)	0.067
Symptom relief within 1 hour (n, %)	2 (8.3)	1 (1.7)	0.126	0 (0.0)	-	0 (0.0)	-
Admission to hospital (n, %)	20 (83.3)	48 (81.4)	0.722	6 (60.0)	0.090	8 (88.9)	0.492

Abbreviations: PCR, polymerase chain reaction.

[1)] Gastrointestinal symptoms include vomiting, abdominal pain, and diarrhea.

* indicates a P value of < 0.05

Supplementary Table S4. Association of other respiratory virus infections with clinical symptoms, signs, treatments, and outcomes in children experiencing asthma exacerbation.

Clinical symptoms and signs, treatments, outcomes	Virus not detected (n=24)	Parainfluenza virus	Coronavirus OC43/NL63/229E	Bocavirus	Influenza virus	Metapneumovirus
Positive in PCR test (n/N, %)		5/96 (5.2)	4/96 (4.2)	3/96 (3.1)	2/96 (2.1)	2/96 (2.1)
Symptoms and signs						
Dyspnea (n, %)	23 (95.8)	5	3	3	1 (P = 0.010) *	1 (P = 0.010) *
Tachypnea (n, %)	15 (62.5)	4	3	3	1	1
Chest retraction (n, %)	11 (45.8)	4	1	3	1	1
Wheezing (n, %)	24 (100.0)	4 (P = 0.026) *	3 (P = 0.010) *	3	2	1 (P < 0.001) *
Gastrointestinal symptoms [1)] (n, %)	2 (8.3)	0	0	0	0	0
Oxygen supplementation (n, %)	10 (41.7)	1	2	1	1	0
Nasal cannula (n, %)	9 (37.5)	1	2	1	1	0
Mask (simple, reservoir bag, venturi) (n, %)	2 (8.3)	0	0	0	0	0
High-flow nasal cannula (n, %)	0 (0.0)	0	0	0	0	0
Systemic steroid administration (n, %)	18 (75.0)	4	3	3	2	0
Symptom relief within 1 hour (n, %)	2 (8.3)	0	0	0	1	0
Admission to hospital (n, %)	20 (83.3)	3	3	0	0	2

Abbreviations: PCR, polymerase chain reaction.

[1)] Gastrointestinal symptoms include vomiting, abdominal pain, and diarrhea.

* indicates a P value of < 0.05

Supplementary Table S5. Logistic regression analysis of the association between respiratory virus and clinical symptoms, signs, treatments, and outcomes in children with asthma exacerbations (N = 96)

Clinical symptoms and signs, treatments, outcomes	Rhinovirus				Adenovirus				RSV			
	cOR, 95% CI	P value	aOR[1)], 95% CI	P value	cOR, 95% CI	P value	aOR[1)], 95% CI	P value	cOR, 95% CI	P value	aOR[1)], 95% CI	P value
Symptoms and signs												
Dyspnea (n, %)	3.455 (0.600–19.893)	0.165	2.762 (0.383–19.936)	0.314	0.556 (0.058–5.296)	0.609	0.251 (0.019-3.289)	0.293	0.488 (0.051-4.705)	0.535	0.375 (0.031–4.492)	0.439
Tachypnea (n, %)	3.009 (1.130–8.013)	0.027*	4.457 (1.497–13.268)	0.007*	1.212 (0.238–6.176)	0.817	1.535 (0.282-8.342)	0.620	0.559 (0.128–2.446)	0.440	0.453 (0.095–2.157)	0.320
Chest retraction (n, %)	2.680 (1.144–6.279)	0.023*	3.142 (1.276–7.734)	0.013*	2.880 (0.577–14.374)	0.197	3.185 (0.618–16.407)	0.166	0.291 (0.068–1.244)	0.096	0.264 (0.060–1.162)	0.078
Wheezing (n, %)	0.792 (0.069–9.051)	0.851	0.816 (0.047–14.311)	0.889	0.214 (0.018–2.603)	0.227	0.051 (0.002–1.331)	0.074	-	-	-	-
Gastrointestinal symptoms[2)] (n, %)	0.938 (0.149–5.893)	0.945	0.908 (0.139–5.941)	0.920	-	-	-	-	-	-	-	-
Oxygen supplementation (n, %)	1.556 (0.680–3.560)	0.296	1.580 (0.674–3.705)	0.293	4.600 (0.923–22.930)	0.063	5.574 (1.075–28.897)	0.041*	0.467 (0.110–1.986)	0.302	0.477 (0.110–2.064)	0.322
Nasal cannula (n, %)	1.517 (0.661–3.484)	0.326	1.616 (0.682–3.827)	0.276	5.297 (1.062–26.428)	0.042*	6.984 (1.329–36.712)	0.022*	0.536 (0.126–2.280)	0.398	0.531 (0.122–2.313)	0.399
Mask (simple, reservoir bag, venturi) (n, %)	0.614 (0.083–4.558)	0.633	0.555 (0.680–4.507)	0.582	3.074 (0.289–32.73	0.352	3.848 (0.317–46.73	0.290	-	-	-	-

					3)		4)					
High-flow nasal cannula (n, %)	-	-	-	-	-	-	-	-	-	-	-	-
Systemic steroid administration (n, %)	3.060 (1.109-8.440)	0.031*	3.065 (1.087–8.638)	0.034*	1.059 (0.207-5.427)	0.945	1.145 (0.217–6.055)	0.873	0.282 (0.068–1.168)	0.081	0.294 (0.070–1.235)	0.095
Symptom relief within 1 h (n, %)	0.195 (0.020–1.954)	0.165	0.153 (0.014–1.675)	0.124	-	-	-	-	-	-	-	-
Admission to hospital (n, %)	1.204 (0.434–3.341)	0.722	1.270 (0.436–3.698)	0.661	0.317 (0.080–1.263)	0.103	0.250 (0.057–1.100)	0.067	2.087 (0.245–17.784)	0.501	2.049 (0.234–17.940)	0.517

Abbreviations: PCR, polymerase chain reaction.

1) Adjusted by sex, allergy history, familial allergy history

2) Gastrointestinal symptoms include vomiting, abdominal pain, and diarrhea.

* indicates a P value of < 0.05

Disclaimer/Publisher's Note: The statements, opinions and data contained in all publications are solely those of the individual author(s) and contributor(s) and not of MDPI and/or the editor(s). MDPI and/or the editor(s) disclaim responsibility for any injury to people or property resulting from any ideas, methods, instructions or products referred to in the content.
